# The prevalence of virulence determinants in methicillin-resistant *Staphylococcus aureus* isolated from different infections in hospitalized patients in Poland

**DOI:** 10.1038/s41598-022-09517-x

**Published:** 2022-03-31

**Authors:** Barbara Kot, Małgorzata Piechota, Andrzej Jakubczak, Magdalena Gryzińska, Małgorzata Witeska, Agata Grużewska, Katarzyna Baran, Paulina Denkiewicz

**Affiliations:** 1 Institute of Biological Sciences, Faculty of Exact and Natural Sciences, Siedlce University of Natural Sciences and Humanities, 14 Bolesława Prusa Str., 08-110 Siedlce, Poland; 2 Institute of Biological Basis of Animal Production, Faculty of Animal Sciences and Bioeconomy, University of Life Sciences in Lublin, Akademicka 13, 20-950 Lublin, Poland; 3 Institute of Agriculture and Horticulture, Faculty of Agrobioengineering and Animal Husbandry, Siedlce University of Natural Sciences and Humanities, 12 Bolesława Prusa Str., 08-110 Siedlce, Poland

**Keywords:** Microbiology, Health care

## Abstract

Methicillin-resistant *Staphylococcus aureus* (MRSA) is responsible for hard-to-treat infections. The presence of 19 virulence genes in 120 MRSA isolates obtained from hospitalized patients and genetic relationships of these isolates were investigated. The *eno* (100%) and *ebps* (93.3%) genes encoding laminin- and elastin binding proteins, respectively, were ubiquitous. Other adhesion genes: *fib* (77.5%), *fnbB* (41.6%), *bbp* (40.8%), *cna* (30.8%) encoding proteins binding fibrinogen, fibronectin, bone sialoprotein and collagen, respectively, and *map*/*eap* (62.5%), encoding Eap, were also frequent. The *etB* and *etD* genes, encoding exfoliative toxins, were present in 15.6% and 12.5% isolates, respectively. The *splA*, *splE* and *sspA*, encoding serine protease were detected in 100%, 70.8% and 94.2% isolates, respectively. The *tst* gene, encoding toxic shock syndrome toxin-1 was found in 75% isolates. The *cna*, *map/eap* and *tst* genes were the most common in wound isolates and much less common in blood isolates. We identified 45 different *spa* types, t003 (21.7%) and t008 (18.8%) being the most common. The t003 was the most frequent among isolates from the respiratory tract (35.5%), while t008 in blood isolates (40%). Identification of virulence factors of MRSA is important for evaluation of pathogen transmission rate and disease development.

## Introduction

Methicillin-resistant *Staphylococcus aureus* (MRSA) cause hard-to-treat infections in various patient populations, and thus is a serious health problem. Treatment of MRSA infections is a significant financial burden for medical institutions^1^. The gene *mecA* in MRSA genome, encodes an enzyme responsible for crosslinking the peptidoglycans in the bacterial cell wall called penicillin-binding protein 2a (PBP2a). Low affinity PBP2a to β-lactams results in resistance to β-lactam antibiotics including penicillins, cephalosporins (except for ceftaroline and ceftobiprole), and carbapenems^2^. Multidrug-resistant (MDR) MRSA isolates are often resistant to commonly used antibiotic groups, such as aminoglycosides, fluoroquinolones, macrolides, tetracycline and chloramphenicol^3,4^.

MRSA is responsible for skin and surgical wound infections but also may infect different parts of the body including lower respiratory tract, cause bloodstream infection (BSI) and toxin-mediated syndromes as well as life-threatening diseases^5–7^. The studies show that the proportion of MRSA among all *S. aureus* isolates is between 13 and 89%^8,9^. MRSA remains a prominent pathogen with persistently high mortality^10^. The mortality rate of *S. aureus* bacteremia is around 20–30%^11^. A European report from a Finnish Hospital Infection Program showed that *S. aureus* ranked among the top three organisms causing bloodstream infections^12^. MRSA are widely spread in various countries, in hospital environments, in community and livestock^13,14^. MRSA infections are routinely detected in hospitalized patients (HAIs, health care-associated infections), also in high-income countries^6,11^. Infections caused by MRSA isolates lead to prolonged hospital stay, increased mortality, especially in patients with underlying diseases such as malignancy or chronic pulmonary diseases^15^. The virulence factors produced by MRSA can be divided into different groups, including degradative enzymes, adhesins, and superantigenic toxins. The infective process requires participation of numerous virulence determinants. First step of staphylococcal infection is the attachment of bacterial cells to the host tissues. *S. aureus* produces the surface-exposed proteins (MSCRAMMs—microbial surface components recognizing adhesive matrix molecules)^16^. Using these MSCRAMMs *S. aureus* can bind to one or more host extracellular matrix factors including laminin, elastin, fibrinogen, fibronectin and collagen^17^. Other protein expressed by *S. aureus* is extracellular adherence protein (Eap) belonging to SERAMs (secretable expanded repertoire adhesive molecules) can bind to various glycoproteins of extracellular matrix (ECM), such as fibronectin, fibrinogen, sialoprotein and some collagens^18^. According to Hussain et al.^19^, this protein is also involved in adhesion of *S. aureus* to fibroblasts as well as in bacteria internalization. *S. aureus* produce also proteases being important virulence factors that can cleave host proteins and allow the transition of MRSA cells from an adhesive to an invasive phenotype. Bacterial skin invasion is facilitated by exfoliative toxins, whereas enterotoxins and the toxic shock syndrome toxin-1, as superantigens suppress the host immune response and promote the persistence of *S. aureus* in host organism^20^. Evaluation of the virulence potential seems to be a reliable method of predicting the behaviour of these bacteria in host organisms, which is very important because it allows to predict the development and course of an infection. The role of virulence factors in the pathogenesis of MRSA may vary with the type of infection and the patient's health status. In Poland, only few works included the virulence potential of MRSA isolated from humans and these studies concerned the population of MRSA isolates from one specific clinical materials^21,22^ or from residents and personnel of nursing home^23^. In this study, our aim was to examine the presence of 19 important virulence genes encoding adhesins, proteases and superantigenic toxins in 120 of MRSA isolates from different clinical materials and to determine the genetic virulence profiles specific for MRSA isolates causing different infections in hospitalized patients during 2015–2017 in hospitals of Masovian district in Poland. We also determined clonal diversity of investigated MRSA isolates by *spa*-typing.

## Results

**Figure 1:**
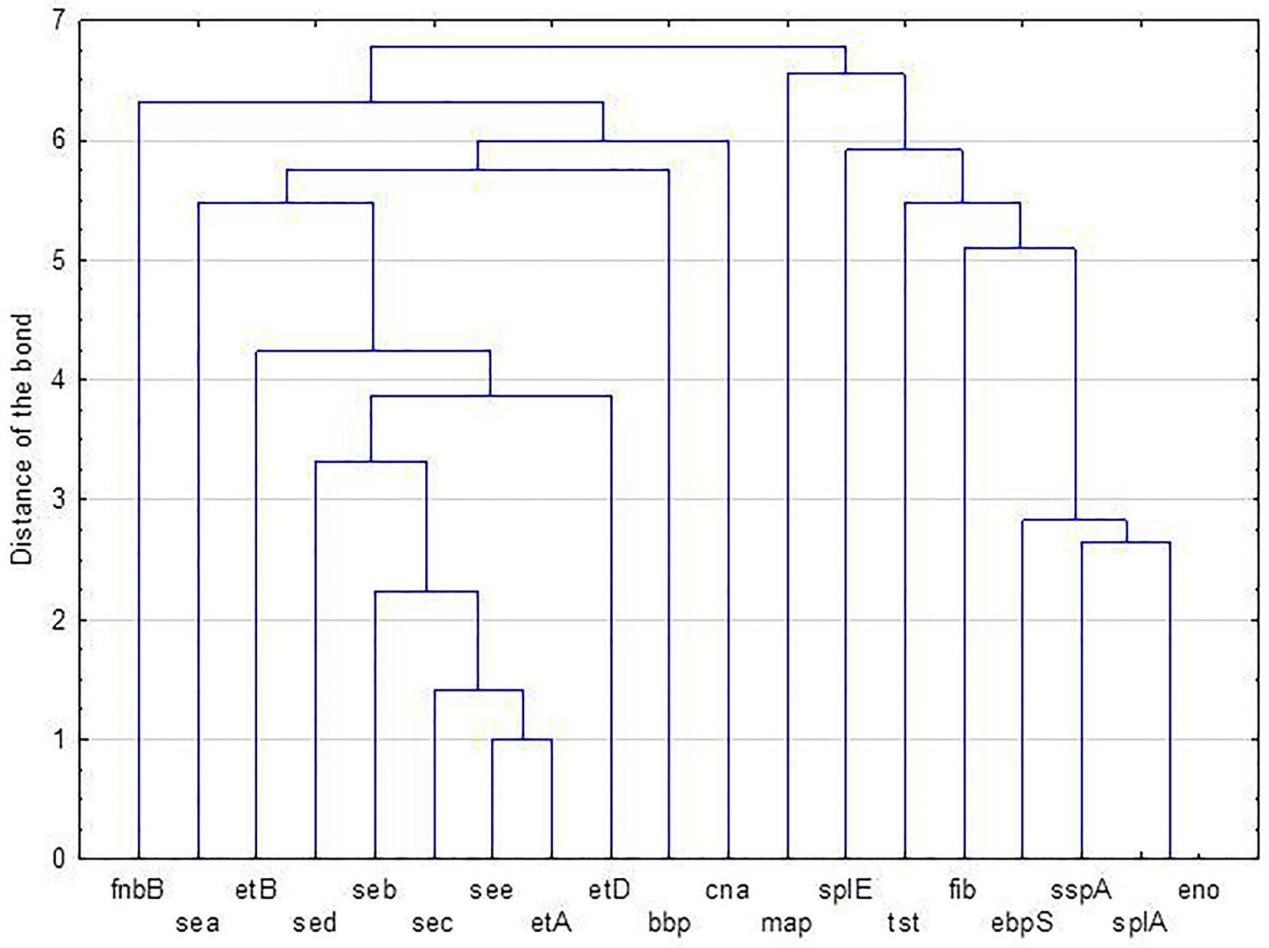
The groups of genes encoding adhesins, proteases and superantigenic toxins in MRSA isolated from patients hospitalized in 2015–2017 created based on the cluster analysis. *fnbB*, *bbp*, *cna*, *fib*, *ebpS*, *eno*—genes encoding adhesins such as fibronectin B-, bone sialoprotein-, collagen-, fibrinogen-, elastin-, laminin-binding proteins, respectively; *map*—gene encoding Eap (extracellular adherence protein); *etA*, *etB*, *etD*—genes encoding exfoliative toxins; *splA*, *sspA*, *splE*—genes encoding serine proteases; *sea*, *seb*, *sec*, *sed*, *see*—genes encoding the enterotoxins A to E; *tst*—gene encoding the toxic shock syndrome toxin-1.

The frequency of 19 genes encoding virulence factors investigated by PCR in 120 MRSA isolates is shown in Table 1. The results are presented for three groups created according to related genes: the adhesin genes, the protease genes and the superantigenic toxin genes encoding the enterotoxins and the toxic shock syndrome toxin-1. The *eno* gene encoding laminin binding protein was identified in all MRSA isolates. Among the remaining genes encoding adhesins, the *ebps* (93.3%) and *fib* (77.5%) encoding elastin and fibrinogen binding proteins, respectively, were the most frequently identified in the investigated MRSA isolates. The *map/eap* gene encoding Eap (62.5%) was also frequently identified. The *fnbB* and *bbp* genes encoding fibronectin and bone sialoprotein binding proteins, were found in more than 40% of isolates. The *cna* gene encoding collagen binding protein was one of the less frequently identified because it was found in only in over 30% of the isolates (Table 1). In a group of three investigated serine protease-encoding genes, *splA* gene was identified in all MRSA isolates. The *sspA* and *splE* genes were present in 94.2 and 70.8% investigated MRSA isolates, respectively. The *etB* and *etD* genes encoding exfoliative toxins were present in the genomes of 15 and 12.5% isolates, respectively. Among the genes encoding classical enterotoxins, the *sea* gene was the most frequently identified (25%). The *tst* gene, encoding the toxic shock syndrome toxin-1, was harboured by 75% isolates (Table 1). Cluster analysis of investigated genes showed that adhesin encoding genes (*eno*, *ebps*) and serine protease-encoding genes (*sspA*, *splA*) belonged to one group of genes that occurred most frequently in this population of MRSA isolates. While, the genes encoding exfoliative toxins (*etB*, *etD*) and enterotoxin-coding genes (*seb*, *sec*, *sed*, *see*) were rarely detected in MRSA isolates (Fig. 1). MRSA isolates differed in the presence of virulence genes. The most common virulotypes were *eno*, *ebpS*, *fib*, *map/eap*, *splA*, *sspA*, *tst* (6.7%), *eno, ebpS*, *cna, fnbB, fib*, *bbp*, *map/eap*, *splA*, *splE*, *sspA*, *tst*, *sea* (2.5%) and *eno, ebpS*, *fnbB, fib*, *bbp*, *map/eap*, *splA*, *splE*, *sspA*, *tst*, *sea* (2.5%) (Table 2). In case of isolates from respiratory tract and nose, the most frequent combination of adhesin genes was *eno*, *ebps*, *fib*, *map/eap*. In genome of isolates from anus and blood, the most frequent adhesin genes were *eno*, *ebps*, *fib*, while *eno*, *ebps*, *cna*, *fnbB*, *fib*, *bbp*, *map/eap* genes were frequently detected in isolates from wounds (Table S1). The simultaneous presence of genes encoding serine proteases (*splA*, *splE*, *sspA*) was the most frequently detected in the genomes of isolates from wound, anus and respiratory tract (Table S2). The *tst* and *sea* genes encoding toxins was the most frequent in the investigated MRSA isolates (19.2%) (Table S3). The presence of *cna* gene was the most common in MRSA isolates from wound (53.3%) (Table 3) and it was highly significantly more frequent than in isolates from respiratory tract (18.8%) (p = 0.0016) and in isolates from blood (9.1%) (p = 0.0119). The *fib* gene was present in all isolates from anus (Table 3). This gene was identified significantly less frequently in isolates from wounds (70%) (p = 0.019) than in isolates from anus. The frequency of *fnbB* gene in MRSA isolates obtained from different clinical materials did not differ significantly. The *ebps* gene was detected in all isolates from anus and blood, while the frequency of the *bbp* gene in various groups of isolates did not differ significantly. The *map/eap* gene was the most often identified among wound isolates (73.3%). Among the genes encoding exfoliative toxins, *etB* gene was the most frequently identified in isolates from respiratory tract (20.8%). There were no significant differences in the frequency of the *etD* gene. The *splE* gene was significantly more frequently detected in wound isolates (80%) than in blood isolates (45.5%) (p = 0.0336). Whereas, the *sea* gene was significantly less frequently detected in wound isolates (6.7%) than in respiratory tract isolates (29.2%) (p = 0.017). The presence of *tst* gene was shown in all nose isolates (Table 3). Moreover, this gene was significantly more frequent in wound isolates (90%) than in isolates from respiratory tract (68.8%) (p = 0.0310) and blood (54.5%) (p = 0.012). The *spa* typing of 106 MRSA isolates showed the presence of 45 different *spa* types (Table 4). The most common *spa* types were t003 (23 isolates, 21.7%), t008 (20 isolates, 18.8%), t4474 (5 isolates, 4.7%) and t5224 (5 isolates, 4.7%). t003 *spa* type was the most frequently detected among isolates from respiratory tract (35.5%), while t008 *spa* type was the most frequently present in isolates from blood (40%). The frequency of the most common *spa* types (t003 and t008) in isolates from patients hospitalized in Siedlce and Warsaw did not differ significantly.

**Table 1:** The prevalence of virulence-associated genes in MRSA isolates from patients hospitalized in 2015–2017.

Virulence-associated genes	No. (%) of isolates with gene
**Adhesins**	
*cna*	37 (30.8)
*fib*	93 (77.5)
*fnbB*	50 (41.6)
*ebps*	112 (93.3)
*bbp*	49 (40.8)
*eno*	120 (100)
*map/eap*	75 (62.5)
**Proteases**	
*etA*	0 (0.0)
*etB*	18 (15.0)
*etD*	15 (12.5)
*splA*	120 (100)
*splE*	85 (70.8)
*sspA*	113 (94.2)
**Superantigenic toxins**	
*sea*	30 (25.0)
*seb*	5 (4.2)
*sec*	3 (2.5)
*sed*	11 (9.2)
*see*	1 (0.8)
*tst*	90 (75.0)

**Table 2:** The most frequent virulotypes in MRSA isolates from different clinical specimens.

Virulotypes	Source (No. of isolates)	*spa* type (n)	No. of isolates (%)
*eno*,*ebpS*,*fib*,*map/eap*,*splA*,*sspA*,*tst*	Respiratory tract (6)	t003 (4), t17775 (1), t1842 (1)	8 (6.7)
	Nose (2)	t003 (1), t901 (1)	
*eno,ebpS*,*can,fnbB,fib*,*bbp*,*map/eap*,*splA,splE,sspA*,*tst*,*sea*	Wound (2)	t2944, t899	3 (2.5)
	Anus (1)	t5224	
*eno,ebpS*,*fnbB, fib*,*bbp*,*map/eap*,*splA*,*splE*,*sspA*,*tst*,*sea*	Respiratory tract (2)	t008 (2)	3 (2.5)
	Anus (1)	t6665

**Table 3:** The prevalence of virulence-associated genes in MRSA isolates from different clinical specimens.

Genes	Source of isolation					
	Respiratory tract, n = 48 (%)	Wound, n = 30 (%)	Anus, n = 15 (%)	Blood, n = 11 (%)	Nose, n = 8 (%)	Other, n = 8 (%)
**Adhesins**						
*cna*	9 (18.8)	16 (53.3)	5 (33.3)	1 (9.1)	3 (37.5)	3 (37.5)
*fib*	39 (81.0)	21 (70.0)	15 (100)	9 (81.8)	7 (87.5)	6 (75.0)
*fnbB*	19 (40.0)	15 (50.0)	5 (33.3)	4 (36.4)	2 (25.0)	5 (62.5)
*ebps*	44 (92.0)	29 (96.7)	15 (100)	11 (100)	7(87.5)	6 (75.0)
*bbp*	15 (31.0)	16 (53.3)	9 (60.0)	4 (36.4)	2 (25.0)	3 (37.5)
*eno*	48 (100)	30 (100)	15 (100)	11 (100)	8 (100)	8 (100)
*map/eap*	30 (63.0)	22 (73.3)	10 (66.6)	4 (36.4)	5 (62.5)	4 (50.0)
**Proteases**						
*etA*	0	0	0	0	0	0
*etB*	10 (20.8)	5 (16.7)	0	1 (9.1)	1 (12.5)	1 (12.5)
*etD*	9 (18.8)	4 (13.0)	3 (20.0)	0	0	2 (25.0)
*splA*	48 (100)	30 (100)	15 (100)	11 (100)	8 (100)	8 (100)
*splE*	33 (68.8)	24 (80.0)	11 (73.3)	5 (45.5)	5 (62.5)	7 (87.5)
*sspA*	45 (93.8)	28 (93.0)	14 (93.3)	11 (100)	7 (87.5)	8 (100)
**Toxins**						
*sea*	14 (29.2)	2 (6.7)	4 (26.6)	1 (9.1)	2(25.0)	2 (25.0)
*seb*	1 (2.0)	1 (3.3)	0	0	0	1 (12.5)
*sec*	2 (4.2)	1 (3.3)	0	0	0	0
*sed*	5 (10.4)	3 (10.0)	1 (6.6)	2 (18.2)	0	0
*see*	1 (2.0)	0	0	0	0	0
*tst*	33 (68.8)	27 (90.0)	11 (73.3)	6 (54.5)	8 (100)	6 (75.0)

**Table 4:** Occurrence of the *spa* types among MRSA isolates from different clinical specimens.

*spa* type	Source of isolation						
	Respiratory tract, n = 45 (%)	Wound, n = 23 (%)	Anus, n = 13 (%)	Blood, n = 10 (%)	Nose, n = 8 (%)	Other, n = 7 (%)	Total n = 106 (%)
t003	16 (35.5)	2 (8.7)	3 (23.1)	–	1 (7.7)	1 (14.3)	23 (21.7)
t008	10 (22.2)	2 (8.7)	–	4 (40)	2 (25)	2 (28.6)	20 (18.8)
t4474	2 (4.4)	2 (8.7)	1 (7.7)	–	–	–	5 (4.7)
t5224	–	2 (8.7)	3 (23.1)	–	–	–	5 (4.7)
t3592	–	1 (4.3)	–	–	1 (7.7)	1 (14.3)	3 (2.8)
t1079	2 (4.4)	–	–	–	–	–	2 (1.9)
t1227	–	2 (8.7)	–	–	–	–	2 (1.9)
t276	–	1 (4.3)	1 (7.7)	–	–	–	2 (1.9)
t012	1 (2.2)	1 (4.3)	–	–	–	–	2 (1.9)
t1806	1 (2.2)	1 (4.3)	–	–	–	–	2 (1.9)
t11766	–	–	2 (15.4)	–	–	–	2 (1.9)
t899	–	1 (4.3)	1 (7.7)	–	–	–	2 (1.9)
t3069	–	–	–	2 (20)	–	–	2 (1.9)
t8746	2 (4.4)	–	–	–	–	–	2 (1.9)
t901	–	–	–	–	2 (25)	–	2 (1.9)
t837	1 (2.2)	–	–	–	–	–	1 (0.9)
t6447	1 (2.2)	–	–	–	–	–	1 (0.9)
t627	1 (2.2)	–	–	–	–	–	1 (0.9)
t1777	1 (2.2)	–	–	–	–	–	1 (0.9)
t2379	1 (2.2)	–	–	–	–	–	1 (0.9)
t034	1 (2.2)	–	–	–	–	–	1 (0.9)
t6401	1 (2.2)	–	–	–	–	–	1 (0.9)
t4657	1 (2.2)	–	–	–	–	–	1 (0.9)
t6506	1 (2.2)	–	–	–	–	–	1 (0.9)
t7784	–	1 (4.3)	–	–	–	–	1 (0.9)
t437	–	1 (4.3)	–	–	–	–	1 (0.9)
t16934	–	1 (4.3)	–	–	–	–	1 (0.9)
t026	–	1 (4.3)	–	–	–	–	1 (0.9)
t037	–	1 (4.3)	–	–	–	–	1 (0.9)
t2944	–	1 (4.3)	–	–	–	–	1 (0.9)
t870	–	1 (4.3)	–	–	–	–	1 (0.9)
t6447	–	–	1 (7.7)	–	–	–	1 (0.9)
t6665	–	–	1 (7.7)	–	–	–	1 (0.9)
t11792	–	–	–	1 (10)	–	–	1 (0.9)
t12426	–	–	–	1 (10)	–	–	1 (0.9)
t5655	–	–	–	1 (10)	–	–	1 (0.9)
t014	–	–	–	1 (10)	–	–	1 (0.9)
t1842	1 (2.2)	–	–	–	–	–	1 (0.9)
t1282	1 (2.2)	–	–	–	–	–	1 (0.9)
t018	–	–	–	–	1 (7.7)	–	1 (0.9)
t1446	–	–	–	–	1 (7.7)	–	1 (0.9)
t002	–	1 (4.3)	–	–	–	–	1 (0.9)
t12250	–	–	–	–	–	1 (14.3)	1 (0.9)
t5160	–	–	–	–	–	1 (14.3)	1 (0.9)
t1209	–	–	–	–	–	1 (14.3)	1 (0.9)

## Discussion

We undertook studies to assess the virulence potential of MRSA isolates from different clinical materials from hospitalized patients because such an analysis is necessary to predict the development and course of infection in the host organism and is important to control it. We also assessed whether MRSA isolates causing infections localized at different sites in the human body differ in the set of pathogenicity-determining genes, and whether the genes encoding virulence factors were associated with the type of infection or with the *spa* type. According to our knowledge, the number of studies on the virulence factors of MRSA isolates from hospitalized patients in Poland is limited and concern small collections of MRSA isolates^22,23^ or only few virulence factors^21^. Among the genes encoding adhesins, the *eno* and *ebps* genes encoding the ability of adhesion to laminin and elastin, respectively, were ubiquitous in the investigated MRSA isolates. The *eno* gene encodes α-enolase, which is able to bind laminin and also functions as a plasminogen receptor. Laminin is a major component of the basal membrane of the vasculature and thus adherence to laminin may contribute to tissue invasion and dissemination of staphylococcal cells by blood to different sites in the host. Besides, α-enolase may cause laminin degradation by activation of plasminogen^24^. Similarly to our results, Kasela et al.^23^ also showed that *eno* gene was present in all MRSA isolates from the residents and personnel of a nursing home in Poland, while Haghi et al.^25^ reported high prevalence of this gene among different clinical isolates. Elastin is a major component of elastic fibres in the extracellular matrix that are present in the skin, lung and blood vessels. Elastin-binding protein of *S. aureus* may be significant mainly in colonisation of injured tissues abundant in elastin^26^. In our study the *ebps* gene was among the most frequently identified genes in the investigated population of MRSA. High frequency of *ebps* gene in MRSA isolates from hospitalised patients indicated that EbpS protein is an important virulence factor of MRSA that binds to elastin and this interaction may promote bacterial colonization and facilitate pathogenesis. In our earlier research^27^ we showed that the expression levels of the *ebps* gene were significantly higher in the first hours of growth under biofilm conditions compared to growth under planktonic conditions, which suggest that the product of this gene is important for the first phase of biofilm growth, in which bacterial cells interact with host extracellular ligands. In the investigated population of MRSA isolates, the *fib* gene encoding fibrinogen binding protein Fib was also frequently identified (77.5%) which is similar to the results (62.2%) obtained by Azmi et al.^28^. Fibrinogen is a glycoprotein present in the blood and it is one of main proteins deposited on implanted biomaterials. The Fib is an important adherence factor responsible for the ability of *S. aureus* to adhere to fibrinogen adsorbed on biomaterials and endothelial cells^29^. Among adhesin genes, the *map/eap* gene encoding a staphylococcal surface protein was present in 62.5% of MRSA isolates. Eap, besides the ability to bind to many different glycoproteins (fibronectin, fibrinogen, sialoprotein and some collagens)^18^ can also form oligomers, and by rebinding to the surface of staphylococcal cells, mediates bacterial agglutination^19^. Damaged tissues in wounds provide a matrix of proteins, including fibrinogen, fibronectin, collagen and albumin to which pathogens may adhere. In our study, the *map/eap* gene was present in many MRSA isolates, but was the most often identified in wound isolates, which indicate that Eap belongs to the group of adhesins important for wound bed colonisation and biofilm formation in wounds. In this study, similarly as the *map/eap* gene, the *cna* gene was also the most frequently identified in wound isolates. Collagen is present in tissues as the structural support but in a wounded tissue becomes available to adhering bacterial cells. The presence of *cna* gene mainly in wound isolates indicate that the product of this gene is essential for the occupation of wound environment and development of infection. Other authors also confirmed that adhesion of *S. aureus* to collagen promoted infection and the initiation of biofilm formation^30^. The ability of adhesion to fibronectin is important in the disease development process, because fibronectin is a ubiquitous host protein present in soluble form in the blood and in fibrillar form in cellular matrices^31^. In our study, we found no relationship between the presence of the *fnbB* gene and the source of MRSA isolation. Contrary to the results obtained by Szczuka et al.^32^ who did not show the presence of *bbp* gene in *S. aureus* from hospitalised patients in Poland, we identified this gene in a high percentage of isolates (40.8%). In our study, the frequency of *bbp* gene encoding the bone sialoprotein binding protein did not differ significantly among the groups of isolates, although the studies by other authors suggested that *S. aureus* with *bbp* were significantly associated with haematogenous osteomyelitis or arthritis^33,34^. In our study, none of the isolates was obtained from osteomyelitis or arthritis but we showed that other clinical materials could be an important source of isolates with *bbp* gene that may be probably also responsible for development of other diseases. Vazquez et al.^35^ (2011) showed that human fibrinogen is also a ligand for Bbp which indicated that other host extracellular matrix factors can be a Bbp-mediated *S. aureus* binding site. Campoccia et al.^36^ showed that co-existence of *bbp* and *cna* genes in *S. aureus* which caused orthopaedic implant infections indicate that adhesins encoded by these genes may act together during the colonization of an orthopaedic implant. In our study, we obtained MRSA isolates, except for blood isolates, in which both the *cna* and *bbp* genes were present simultaneously. In our research, we showed that the *splE* was significantly more frequently detected in wound isolates than in blood isolates isolates. The Spl proteases are a group of six serine proteases that are encoded on the pathogenicity island and are unique to *S. aureus*. Paharic et al.^37^ showed that SplA may promote invasion and spreading of *S. aureus* by removing mucin 16 from epithelial cells. Since mucin 16 is found on ocular, airway, and female reproductive tract epithelial cells, this function could facilitate infection of multiple body sites. The *etA*, *etB* and *etD* genes encode exfoliative toxins (ETs), which are unique serine proteases and are related to human infections^38^. ETA and ETB selectively recognize and hydrolyze desmosomal proteins in the skin and are responsible for localized epidermal infections such as bullous impetigo and generalized diseases like staphylococcal scalded skin syndrome (SSSS), predominantly in children^39^. ETA is encoded by the chromosomally located *etA* gene, while ETB by *etB* gene located on a large plasmid^40^. In the present study, we did not obtain MRSA isolates with *etA* gene, which is consistent with the results shown by other Polish authors^23^. Whereas, *etB* gene was present in 15% of MRSA isolates and this frequency was higher than study conducted in Turkey by Demir et al.^41^ who showed that this gene was present in 9.2% of *S. aureus* isolates. ETD mediates intra-epidermal cleavage through the granular layer of the epidermis of newborn mice and induces epidermal blisters. The *etD* gene is encoded in a pathogenicity island on the chromosome^42^. It was shown that *S. aureus* strains that produced ETD extracellularly were isolated mainly from other sources of infections and not from patients with bullous impetigo or staphylococcal scalded-skin syndrome. This indicates that ETD might play a role in a broader spectrum of bacterial infections than previously considered^42^. This is in line with our results because MRSA isolates with the *etD* gene were isolated from different clinical materials and we did not show significant differences in the frequency of *etD* in isolates from different sources. The *tst* gene encodes the toxin of toxic shock syndrome which mediates acute life threatening infections by stimulating release of cytokines such as IL-1, IL-2, TNF-α and others^43^. The presence of MRSA able to produce a toxic-shock syndrome toxin may increase the risk of the occurrence of more severe symptoms during infection. El-baz et al.^44^ showed the presence of this gene in about 66% of MRSA isolates, while Eftekhar et al.^45^ in 51.4%. In our research, this gene was present in all isolates from nose, while in isolates from blood and respiratory tract was detected significantly less often than in wound isolates. Our results are in line with those obtained by other authors who reported high frequency of this gene in MRSA isolates from wound and blood samples^45^ or respiratory tract^46^. Among genes encoding staphylococcal enterotoxins, we found the presence of *sea* gene in 25% of MRSA isolates, while remaining investigated enterotoxin genes were detected in a few isolates. The study of Aggarwal et al.^47^ concerning *S. aureus* isolated from a variety of nosocomial infections, showed that in this population 30.2% of strains were positive for *sea* gene.

Epidemiological monitoring of MRSA infection is essential to control the occurrence and spread of epidemic clones within and between hospitals. In our study, we used *spa* typing method to distinguish MRSA isolates. This method is based on the sequencing of short repetitive sequences of the polymorphic X region from the gene encoding protein A^48–50^. Main advantage of *spa* typing is the portability of sequence data which simplifies the sharing of information between laboratories and facilitates the creation of the large-scale database for the study of global and local epidemiology^51^. In addition, this method can be used to study the molecular evolution and genetic variability of *S. aureus* and enables the correct assignment of isolates to phylogenetic lines^48^. Therefore, this method can also be used to differentiate MRSA responsible for infection outbreaks in hospitals, and the results obtained can be compared between laboratories. Many studies showed that the *spa* types are area specific^52,53^. In our study, *spa* typing of MRSA isolates showed high diversity of isolates which indicates that these isolates have emerged from outside the hospitals. Nevertheless, t003 (21.7%), t008 (18.8%), t4474 and t5224 (4.7%) dominated among 45 different *spa* types. *Spa* type t003 corresponds to the epidemic clone isolated in the USA and in many European countries^51^. Several authors showed that *spa* type t003 is the most predominant among MRSA isolates in hospitals of Southern Poland^21,22^ and of Northern Poland^54^. Our results showed that *spa* type t003 is also the most predominant among MRSA isolates in hospitals of Central Poland. The results also revealed that among MRSA isolates *spa* type t008 belonged to the most frequent which was in line with the results of Wiśniewska et al.^54^, who investigated clinical MRSA in Northern Poland.

## Conclusion

MRSA isolates responsible for variously localized infections in patients hospitalized in Central Poland showed high variation in terms of the presence of genes encoding important virulence factors. Irrespective of the source of isolation, the genes encoding laminin-, elastin- and fibrinogen binding proteins, serine proteases and toxic shock syndrome toxin-1 were the most commonly identified virulence factors. The results also showed that some genes: *cna* gene encoding collagen binding protein and *map/eap* encoding Eap were more common in wound isolates compared to the other sources. The obtained results showed genetic diversity of the analyzed group of MRSA isolates, which indicates that they originated from an outside hospital environment and did not show a relationship. The identification of pathogenic factors of MRSA isolates is important for evaluation of pathogen transmission rate, disease development rate and its severity. Therefore, analysis of potential virulence of MRSA provides information important for choice of appropriate methods of prevention and treatment of MRSA infections. Further studies should focus on development of factors preventing adhesion and biofilm formation by MRSA which would considerably increase treatment effectiveness.

## Material and methods

### MRSA isolates

A total of 120 MRSA isolates from human clinical materials such as swabs from wound (30), anus (15), nose (8), blood samples (11), respiratory tract (48), and other samples: swabs from tracheostomy tube, endotracheal tube and catheter and urine (8) were used in this study. The MRSA isolates were obtained from hospitals in Siedlce (83) and Warsaw (37) (Poland) in 2015–2017 (Table 5). The MRSA isolates were collected as part of routine diagnostic microbiology and came from different patients. The numbers of MRSA isolates obtained in 2015, 2016 and 2017 were 18, 24 and 78, respectively. Identification of isolates as *S. aureus* was confirmed by Gram's staining, positive test for catalase activity and tube test for coagulase. The PCR analysis to amplify the part of the *nuc* gene, encoding a thermostable nuclease specific for *S. aureus* was used to confirm that the isolates belonged to the *S. aureus* species^55^. The presence of the *mecA* gene responsible for resistance against β-lactam antibiotics of these isolates was identified by PCR^3^.

**Table 5:** MRSA isolation sources.

Isolates from hospital in:	Source of isolation					
	Respiratory tract, n = 48	Wound, n = 30	Anus, n = 15	Blood n = 11	Nose, n = 8	Other, n = 8
Siedlce (n = 83)	36	20	10	9	6	2
Warsaw (n = 37)	12	10	5	2	2	6

### DNA isolation

Genomic DNA from bacterial cells was isolated by using the NucleoSpin Microbial DNA (Macherey–Nagel GmbH&Co.KG, Düren, Germany) according to the manufacturer’s protocol. 2.5 µl of the total extracted material from each test sample was used as a template DNA for PCR application.

### Primers and PCR conditions

The primer sequences specific for the genes of adhesins, proteases, gene of toxic shock syndrome toxin-1 and for A, B, C, D and E enterotoxin genes, synthesized at DNA-Gdańsk (Gdańsk, Poland), are listed in Table 6.

**Table 6:** Primers used in PCR.

Gene	Protein	Primer sequences (5′ → 3′)	Length (bp)	References
*cna*	Collagen binding protein	GTCAAGCAGTTATTAACACCAGAC AATCAGTAATTGCACTTTGTCCACTG	423	Tristan et al.^33^
*fib*	Fibrinogen binding protein	CTACAACTACAATTGCCGTCAACAG GCTCTTGTAAGACCATTTTCTTCAC	404	
*fnbA*	Fibronectin binding protein A	GTGAAGTTTTAGAAGGTGGAAAGATTAG GCTCTTGTAAGACCATTTTTCTTCAC	643	
*fnbB*	Fibronectin binding protein B	GTAACAGCTAATGGTCGAATTGATACT CAAGTTCGATAGGAGTACTATGTTC	524	
*ebps*	Elastin binding protein	CATCCAGAACCAATCGAAGAC CTTAACAGTTACATCATCATGTTTATCTTTG	186	
*bbp*	Bone sialoprotein binding protein	AACTACATCTAGTACTCAACAACAG ATGTGCTTGAATAACACCATCATCT	575	
*eno*	Laminin binding protein	ACGTGCAGCAGCTGACT CAACAGCATTCTTCAGTACCTTC	302	
*map/eap*	Broad binding of extracellular matrix proteins	TAACATTTAATAAGAATCAA CCATTTACTGCAATTGT	943–9	Rohde et al.^56^
*etA*	Exfoliative toxin A	TTGTAAAAGGACAAACAAGTGC TTCCCAATACCAACACC	544	Ote et al.^57^
*etB*	Exfoliative toxin B	TTACAAGCAAAAGAATACAGCG GGAAGATTATGTTGTCCGCC	641	
*etD*	Exfoliative toxin D	ACTATCATGTATCAAGGATGGCT CTCCTTTTCCAACATGAATACC	432	
*splA*	Serine protease SplA	GCGGGTGGTACTGGTGTAGT CGTTCCTGTCGATTCAAACA	339	
*splE*	Serine protease SplE	ATGTCGTTGCAGGTATGG CCGTTTCCACCAAAGTGA	409	
*sspA*	Serine V8 protease	GATGCTACGCACGGTGATCC GGTTGGTCATCGTTGGCA	503	
*sea*	Enterotoxin A	CAGCATACTATATTGTTTAAAGGC CCTCTGAACCTTCCCATC	400	Park et al.^58^
*seb*	Enterotoxin B	GTATGGTGGTGTAACTGAGCA TCAATCTTCACATCTTTAGAATCA	351	
*sec*	Enterotoxin C	CTCAAGAACTAGACATAAAAGCTAGG TCAAAATCGGATTAACATTATCC	271	Becker et al.^59^
*sed*	Enterotoxin D	CTAGTTTGGTAATATCTCCTTTAAACG TTAATGCTATATCTTATAGGGTAAACATC	319	
*see*	Enterotoxin E	CAGTACCTATAGATAAAGTTAAAACAAGC TAACTTACCGTGGACCCTTC	178	
*tst*	Toxic shock syndrome toxin-1	TTTTTTATCGTAAGCCCTTTGTTGC CACCCGTTTTATCGCTTGAA	550	Ote et al.^57^

The monoplex PCR for each gene was performed in a 25 µL volume containing 2.5 µL of DNA template, 1 × PCR buffer, 0.2 mM each dATP, dCTP, dGTP and dTTP (Fermentas, Vilnius, Lithuania), the specific primers at 200 nM, and 1 U of RedTag Genomic DNA polymerase (Sigma Aldrich, Steinheim, Germany). The PCR method described by Tristan et al.^33^ was used for detection of genes: *cna*, *fib*, *fnbB*, *ebps*, *bbp*, *eno* encoding adhesins such as collagen-, fibrinogen-, fibronectin B-, elastin-, bone sialoprotein-, laminin-binding protein, respectively. The *map*/*eap* gene was amplified by PCR according to Rohde et al.^56^.

The thermal cycling conditions for detection of genes encoding protease (*etA* and *etB*) included predenaturation at 95 °C for 4 min, 35 cycles of denaturation at 95 °C for 0.5 min, primer annealing at 56 °C for 0.5 min and extension at 72 °C for 1 min. The primer annealing at 59 °C was used for the detection of genes coding the remaining proteases (*etD*, *splA*, *splE*, *sspA*). A 5-min extension at 70 °C was performed at the end of the final cycle. PCR for enterotoxin genes was carried out according to the protocol described earlier by Piechota et al.^60^. The *tst* gene coding the toxic shock syndrome toxin-1 was detected in accordance with the method described by Ote et al.^57^. Amplifications were carried out in the Eppendorf Mastercycler Nexus Gradient (Hamburg, Germany). A negative control was composed of all components of the PCR mixture, except for a template DNA. The positive controls included the genomic DNA from the MRSA isolates in which the presence of the searched genes was previously found in the genome. These controls were included in each test run. The PCR products were analysed by electrophoresis in 1.5% agarose gels stained with ethidium bromide. Molecular size markers (Sigma-Aldrich) were also run for verification of product size. The gel was electrophoresed in a 2 × Tris–borate buffer at 70 V for 1.5 h. The PCR amplicons were visualized using UV light (Syngen Imagine, Syngen Biotech, Wrocław, Poland).

### Spa-typing

The highly polymorphic X region of *Staphylococcus* protein A (*spa*) gene was amplified by PCR according to Szweda et al.^61^. Amplified products were purified, and both standards were sequenced using an ABI PRISM® 3100 Genetic Analyzer (Applied Biosystems Hitachi, Waltham, Massachusetts, USA). The nucleotide sequences were analysed to assign the isolates to various types using the *spa* typing website Ridom SpaServer (http://spaserver.ridom.de).

### Data analyses

Chi-squared statistics in Statistix 11.0 (Analytical Software, Tallahassee, FL, USA) was used for testing the significance of differences in the frequency of presence of each gene between pairs of proportions (percentages) of isolates from various isolation sources.

### Ethical approval

According to Polish law and the regulations of the Scientific Research Ethics Committee established by the Rector's Order No 128/2020, Siedlce University of Natural Sciences and Humanities, no ethical approval was required for this study and all the data were anonymous.

## Supplementary Information

Supplementary Information.
