# Supplementary material for: The prevalence of virulence determinants in methicillin-resistant Staphylococcus aureus isolated from different infections in hospitalized patients in Poland
Source: Sci Rep. 2022 Mar 31;12:5477. doi: 10.1038/s41598-022-09517-x (PMC8971418; doi:10.1038/s41598-022-09517-x)
Supplement: Supplementary file 1 — Supplementary Information. [file 41598_2022_9517_MOESM1_ESM.pdf]

The prevalence of virulence determinants in methicillin-resistant *Staphylococcus aureus* isolated from different infections in hospitalized patients in Poland

Barbara Kot, Małgorzata Piechota, Andrzej Jakubczak, Magdalena Gryzińska, Małgorzata Witeska, Agata Grużewska, Katarzyna Baran, Paulina Denkiewicz

Source (no. of isolates)	Adhesin genes							No. (%) of isolates with gene
	eno	*ebps*	*cna*	*fnbB*	*fib*	*bbp*	*map/eap*	
Respiratory tract (48)	+	+	-	-	+	-	+	12 (25.0)
	+	+	-	-	+	-	-	6 (12.5)
	+	+	-	+	+	-	+	5 (10.4)
	+	+	-	+	+	+	+	4 (8.3)
	+	+	+	-	-	+	+	4 (8.3)
	+	+	-	+	+	-	-	3 (6.2)
	+	+	-	-	+	+	-	2 (4.2)
	+	-	-	+	+	-	-	2 (4.2)
	+	+	-	+	+	-	+	2 (4.2)
	+	+	+	+	-	-	+	2 (4.2)
	+	+	+	+	+	+	-	1 (2.1)
	+	+	-	-	-	+	-	1 (2.1)
	+	+	-	-	-	-	-	1 (2.1)
	+	-	-	-	-	-	-	1 (2.1)
	+	+	+	-	+	+	+	1 (2.1)
	+	-	-	-	+	+	-	1 (2.1)
Wound (30)	+	+	+	+	+	+	+	4 (13.3)
	+	+	-	-	+	+	+	3 (10.0)
	+	+	+	+	-	-	+	3 (10.0)
	+	+	+	-	+	+	+	2 (6.6)
	+	+	+	+	+	-	+	2 (6.6)
	+	+	-	-	+	+	-	2 (6.6)
	+	+	+	+	+	+	-	1 (3.3)
	+	+	-	+	+	-	+	1 (3.3)
	+	+	+	-	+	+	-	1 (3.3)
	+	-	-	-	+	-	+	1 (3.3)
	+	+	-	+	-	+	+	1 (3.3)
	+	+	+	+	-	+	+	1 (3.3)
	+	+	+	-	+	-	+	1 (3.3)
	+	+	-	-	+	-	+	1 (3.3)
	+	+	+	-	+	-	-	1 (3.3)
	+	+	-	-	-	+	+	1 (3.3)
	+	+	-	+	+	-	-	1 (3.3)
	+	+	-	-	+	-	-	1 (3.3)
	+	+	-	+	-	-	+	1 (3.3)
	+	+	-	-	-	-	-	1 (3.3)
Anus (15)	+	+	-	-	+	-	-	3 (20.0)
	+	+	-	-	+	+	+	2 (13.3)
	+	+	+	+	+	+	+	2 (13.3)
	+	+	+	-	-	+	+	2 (13.3)

	+	+	-	+	+	+	+	1 (6.6)
	+	+	+	-	+	+	+	1 (6.6)
	+	+	-	-	+	-	+	1 (6.6)
	+	+	-	+	-	-	-	1 (6.6)
	+	+	-	-	-	+	+	1 (6.6)
	+	+	-	+	+	-	-	1 (6.6)
Blood (11)	+	+	-	-	+	-	-	4 (36.4)
	+	+	-	-	+	+	+	2 (18.2)
	+	+	-	+	+	+	-	2 (18.2)
	+	+	+	+	-	-	+	1 (9.1)
	+	+	-	+	+	-	+	1 (9.1)
	+	+	-	-	-	-	-	1 (9.1)
Nose (8)	+	+	-	-	+	-	+	2 (25.0)
	+	+	+	-	+	-	+	1 (12.5)
	+	+	+	+	+	-	-	1 (12.5)
	+	+	-	-	+	+	+	1 (12.5)
	+	+	+	-	-	+	+	1 (12.5)
	+	-	-	+	+	-	-	1 (12.5)
	+	+	-	-	+	-	-	1 (12.5)
Other (8)	+	+	-	+	+	-	-	3 (37.5)
	+	+	+	+	-	+	+	1 (12.5)
	+	+	+	-	+	+	-	1 (12.5)
	+	+	-	+	+	+	+	1 (12.5)
	+	-	+	-	-	-	+	1 (12.5)
	+	-	-	-	+	-	+	1 (12.5)

Table S1. The combinations of the adhesin genes in MRSA isolated from patients hospitalized in 2015-2017.

Source (no. of isolates)	Protease genes *splA*	*splE*	*sspA*	*etD*	*etB*	*etA*	No. (%) of isolates with gene
Respiratory tract (48)	+	+	+	-	-	-	16 (33.3)
	+	-	+	-	-	-	13 (27.1)
	+	+	+	+	-	-	6 (12.5)
	+	+	+	-	+	-	5 (10.4)
	+	+	-	-	-	-	3 (6.2)
	+	+	+	+	+	-	3 (6.2)
	+	-	+	-	+	-	2 (4.2)
Wound (30)	+	+	+	-	-	-	18 (60.0)
	+	-	+	-	-	-	4 (13.3)
	+	+	+	-	+	-	3 (10.0)
	+	-	+	+	-	-	2 (6.6)
	+	+	-	-	-	-	2 (6.6)
	+	+	+	+	-	-	1 (14.3)
Anus (15)	+	+	+	-	-	-	8 (53.3)
	+	-	+	-	-	-	3 (20.0)
	+	+	+	+	-	-	2 (13.3)
	+	-	+	+	-	-	1 (6.6)
	+	+	-	-	-	-	1 (6.6)
Blood (11)	+	-	+	-	-	-	6 (54.5)
	+	+	+	-	-	-	4 (36.4
	+	+	+	-	+	-	1 (9.1)
Nose (8)	+	+	+	-	-	-	3 (37.5)
	+	-	+	-	-	-	3 (37.5)
	+	+	+	-	+	-	1 (12.5)
	+	+	-	-	-	-	1 (12.5)
Other (8)	+	+	+	-	-	-	4 (50.0)
	+		+	+	-	-	2 (25.0)
	+	+	+	-	+	-	1 (12.5)
	+	-	+	-	-	-	1 (12.5)

Table S2. The combinations of the protease genes in MRSA isolated from patients hospitalized in 2015-2017.

Source (no. of isolates)	Superantigenic toxin genes *tst*	*sea*	*seb*	*sec*	*sed*	*see*	No. (%) of isolates with gene
Respiratory tract (48)	+	-	-	-	-	-	20 (41.6)
	-	-	-	-	-	-	11 (22.9)
	+	+	-	-	-	-	7 (14.6)
	-	+	-	-	-	-	3 (6.2)
	+	+	-	-	+	-	2 (4.2)
	-	+	-	-	+	-	1 (2.1)
	-	-	-	-	+	-	1 (2.1)
	+	-	-	-	+	-	1 (2.1)
	+	-	+	-	-	-	1 (2.1)
	+	-	-	+	-	+	1 (2.1)
Wound (30)	+	-	-	-	-	-	16 (53.3)
	+	+	-	-	-	-	6 (20.0)
	+	-	-	-	+	-	2 (6.6)
	+	-	+	-	-	-	2 (6.6)
	-	-	-	-	-	-	2 (6.6)
	+	-	-	+	+	-	1 (3.3)
	-	+	-	-	-	-	1 (3.3)
Anus(15)	+	-	-	-	-	-	7 (46.6)
	+	+	-	-	-	-	5 (33.3)
	-	-	-	-	-	-	2 (13.3)
	-	-	-	-	+	-	1 (6.7)
Blood (11)	+	-	-	-	-	-	4 (36.4)
	-	-	-	-	-	-	4 (36.4)
	+	+	-	-	-	-	1 (9.1)
	+	-	-	-	+	-	1 (9.1)
	-	-	-	-	+	-	1 (9.1)
Nose (8)	+	-	-	-	-	-	5 (62.5)
	+	+	-	-	-	-	2 (25.0)
	-	-	-	-	-	-	1 (12.5)
Other (8)	+	-	-	-	-	-	3 (37.5)
	+	+	-	-	-	-	2 (25.0)
	-	-	-	-	-	-	2 (25.0)
	+	-	+	-	-	-	1 (12.5)

Table S3. The combinations of superantigenic toxin genes in MRSA isolated from patients hospitalized in 2015-2017.
